# BETTER LIVING THROUGH TECH: TECHNOLOGY-MEDIATED RECREATION AND LONG-TERM CARE FACILITY RESIDENTS’ QUALITY OF LIFE

**DOI:** 10.1093/geroni/igz038.1660

**Published:** 2019-11-08

**Authors:** Ethan A McMahan, Marion Godoy, Abiola Awosanya, Robert Winningham, Charles De Vilmorin, Meaghan McMahon

**Affiliations:** 1 Western Oregon University, Monmouth, Oregon, United States; 2 Responsive Health Management Inc., Toronto, Ontario, Canada; 3 Cedarvale Long Term Care Home, Toronto, Ontario, Canada; 4 Linked Senior, Washington, D.C., United States; 5 MBM Consulting, Washington, D.C., United States

## Abstract

Empirical research on long-term care facility resident engagement has consistently indicated that increased engagement is associated with more positive clinical outcomes and increased quality of life. The current study adds to this existing literature by documenting the positive effects of technologically-mediated recreational programing on quality of life and medication usage in aged residents living in long-term care facilities. Technologically-mediated recreational programming was defined as recreational programming that was developed, implemented, and /or monitored using software platforms dedicated specifically for these types of activities. This study utilized a longitudinal design and was part of a larger project examining quality of life in older adults. A sample of 272 residents from three long-term care facilities in Toronto, Ontario participated in this project. Resident quality of life was assessed at multiple time points across a span of approximately 12 months, and resident engagement in recreational programming was monitored continuously during this twelve-month period. Quality of life was measured using the Resident Assessment Instrument Minimum Data Set Version 2.0. Number of pharmacological medication prescriptions received during the twelve-month study period was also assessed. Descriptive analyses indicated that, in general, resident functioning tended to decrease over time. However, when controlling for age, gender, and baseline measures of resident functioning, engagement in technologically-mediated recreational programming was positively associated with several indicators of quality of life. The current findings thus indicate that engagement in technology-mediated recreational programming is associated with increased quality of life of residents in long-term care facilities.

